# Reconstruction Layout Optimization of Multivariety and Small Batch Workshop in Aerospace Industry

**DOI:** 10.1155/2022/9181865

**Published:** 2022-12-28

**Authors:** Baohai Zhao, Xingyu Jiang, Zhiqiang Tian, Keqiang Chen, Guozhe Yang, Shun Liu, Minghao Wang, Weijun Liu

**Affiliations:** School of Mechanical Engineering, Shenyang University of Technology, Shenyang 110870, China

## Abstract

To cope with the problems of frequent mold changes, long production cycles and serious logistics crossings in workshop of aerospace enterprise. First, a manufacturing cell layout planning method based on the feature bit code domain method and *K*-Means++ is proposed to realize the accurate division of manufacturing cells. Then, a multiobjective optimization method of dynamic reconstruction layout based on improved fruit fly optimization algorithm (IFOA) is proposed to solve the reconstruction layout optimization of the production workshop problem with the optimization objectives of logistics cost, reconstruction cost, loss cost, and cell integrated area. Finally, plant simulation software is applied to simulate the workshop layout before and after optimization. The simulation results show that the logistics cost of the workshop cell layout after optimization is reduced by 8.7%, the utilization rate of the workshop area is improved by 5.2%, and the value-added rate of products is increased by 6.6%, which verifies the effectiveness and feasibility of the proposed model and method.

## 1. Introduction

The production workshop of an aerospace enterprise is a typical multivariety and small batch discrete production workshop, and the products are characterized by many varieties, small batches, many parts, complex production processes, and coexistence of production and development. Most of the existing production workshops of aerospace enterprises still adopt the traditional cluster layout, which makes it hard to meet the continuously increasing variety of aerospace product production in recent years. According to statistics, the logistics cost and production cost wastage caused by an unreasonable workshop layout reaches 20%–50% of the total cost of production system, and the workshop production cost can be effectively reduced by 10%–30% through reasonable planning of workshop layout [1]. Therefore, an effective optimizing method of production workshop layout in the aerospace enterprise is of great practical significance to reduce the comprehensive costs of workshops as well as improve the value-added rate of products.

The multivariety and small batch facility layout problem is a high-dimensional and nonlinear NP-hard problem [2]. Although the existing cell layout in an aerospace enterprise workshop can carry out the normal production of multivariety and small batch products, the cell formation is subjective and poor in accuracy, while the logistics within the cell are extremely chaotic and costly. Thus, it is still a difficult problem to reasonably construct the manufacturing cell and plan the production logistics within the cell. In recent years, a lot of work has been carried out on cell formation and workshop layout from the perspective of modeling optimization.

In a static environment, scholars at home and abroad have done a lot of research studies [3–14]. Sabrina and Menouar [3] proposed a graph-theoretic model based on group technology principles and developed two B&B algorithms to solve the manufacturing cell formation problem. Liu et al. [4] constructed a timed Petri net model based on the functions and connections of each production cell for a discrete production plant and applied FlexSim for simulation optimization, effectively reducing the cross-detour routes of the plant and the idle rate of the equipment, and improved the productivity of the plant. Wu et al. [5] proposed an improved ant colony optimization algorithm for the large-scale factory layout problem, and the safety, geographic, and environmental constraints are considered in the optimization process to achieve the spatial allocation of the factory layout. Liu et al. [12] used a new heuristic algorithm to obtain Pareto-optimal solutions to the problem and proposed a heuristic layout updating strategy and a niche technology to solve the unequal area facility layout problem. Ren et al. [14] developed a methodology for the reconfigurable modular facilities layout problem with alternative process routings, and an integrated mathematical model is proposed to improve production flexibility and minimize material handling costs.

**Figure 1:**
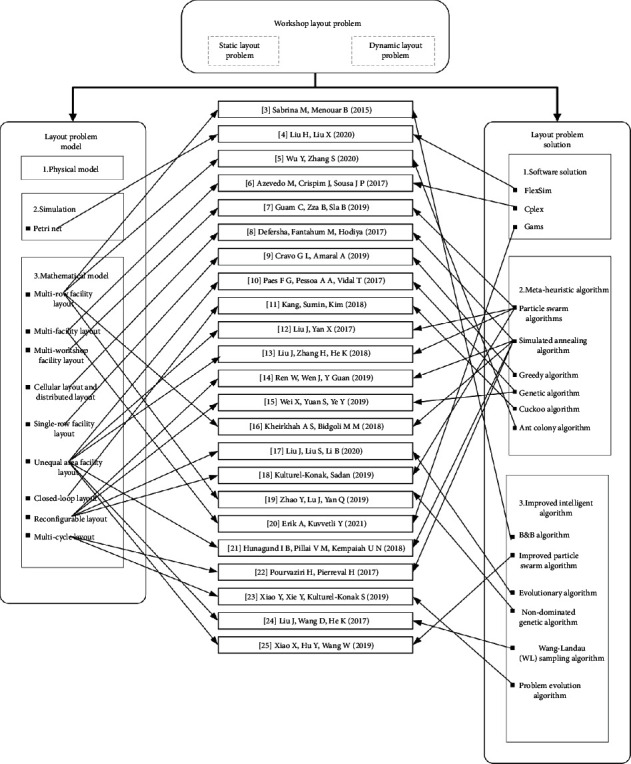
Model and solution of workshop layout problem.

Many research results have been published by domestic and overseas scholars on dynamic layout optimization problems [15–25]. Wei et al. [15] developed a layout model for the reconstruction manufacturing system and applied the chaos genetic algorithm to solve the dynamic facility layout problem. Kheirkhah and Bidgoli [16] proposed an improved simulated annealing algorithm based on graph theory for solving the dynamic facility layout problem by transforming it into the shortest path problem based on practical constraints. The validity of the model and the solution method is verified by numerical experiments. Liu et al. [24] described a model based on the dynamic facility layout problem and combined the Wang–Landau sampling algorithm and some heuristic strategies to solve the unequal area dynamic facility layout problem. Xiao et al. [25] proposed a hybrid robust optimization model for the unequal area dynamic facility layout problem considering the location of pick-up and drop-off points to solve the product demand uncertainty problem. Furthermore, an improved particle swarm optimization algorithm is developed to solve the proposed model. A summary of the research literature on workshop layout issues is shown in Figure 1.

As mentioned previously, extensive results have been achieved in workshop cell construction and layout optimization. However, there are some limitations in the division of manufacturing cells, specific layout of equipment within the cell, and reasonable evaluation of layout schemes in the workshop of aerospace enterprises. At present, most of the research studies on product family construction revolve around grouping techniques, which only start from the product's properties and ignore the actual situation of processing equipment, resulting in an unsatisfactory division of manufacturing cells, and the processing equipment used for the same product family cannot be concentrated in the same cell. After the product family is constructed, it still needs to be artificially adjusted to make the processing equipment concentrated in one manufacturing cell, which cannot guarantee the objectivity of manufacturing cell division. Moreover, the reconstruction layout within the cell can be summarized as a multiobjective dynamic discrete combinatorial optimization problem, for which the large-scale NP complete exact solution cannot be obtained in a limited and reasonable time. This leads to slow convergence of the existing algorithms in solving problems and unsatisfactory multiobjective solutions, which cannot cope with the dynamic changes of the reconstruction layout.

The main contributions of this study can be concluded as follows. (1) A reconstruction workshop layout model with the objectives of minimum logistics costs, reconstruction costs, loss costs, and integrated cell area for multivariety and small batch aerospace enterprise is established. (2) A novel IFOA is proposed to obtain the optimal reconstruction layout plan of aerospace enterprise workshops. (3) The plant simulation is employed to assess the effectiveness of reconstruction layout plans before and after optimization. The rest of this study is organized as follows. The layout planning method is introduced in Section 2. In Section 3, the modeling process of the dynamic reconstruction layout optimization problem is presented.  Section 4 performs a novel IFOA, and a case study is provided to verify the effectiveness of the method in Section 5. In Section 6, the computer simulation and analysis are conducted.  Section 7 summarizes the findings and future works.

## 2. Layout Planning Method

To address the problems of frequent product mold changes and serious logistics crossovers in the production workshops of aerospace enterprises, product families are constructed to reduce the number of product tooling switches and production preparation time, and manufacturing cells are constructed to centralize material flow within the cells to reduce logistics chaos and handling waste. Parts can be categorized into design families, machining families, numerical control families, and management families according to their similar characteristics, among which machining families are applied to grouping processing and facility layout of parts [26]. Existing studies mainly apply grouping techniques to construct product families; however, due to only considering the process similarity and ignoring the product process routes, equipment used for processing the same family of products cannot be concentrated in the same cell, and the product families still need to be artificially adjusted. Therefore, we apply the feature bit code domain method to construct the product design family from the attributes of parts, make preliminary clustering of products, and then apply the *K*-Means++ clustering algorithm combined with the product-equipment matrix to accurately cluster products to construct the product processing family and divide manufacturing cells.

**Figure 2:**
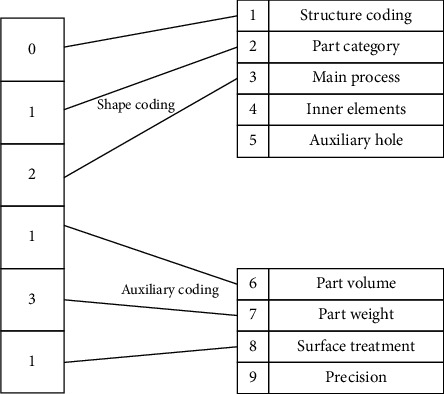
Coding process of feature bit code domain method coding.

The feature bit method encodes the parts by selecting a specific feature bit in the coding system to avoid encoding all the bits in the part coding system. The code domain method is based on the code bit of the part classification coding system, increasing the domain value of the code bit and allowing parts with different coding but similar partial characteristics to be classified into one class. The hybrid method of feature bit and code domain increases the number of components that can be grouped within the group. With the classification of the part features, the requirements for each feature bit are appropriately relaxed, allowing more parts with similar structures and features to be grouped into a product family. The coding process of this hybrid method is shown in Figure 2.

In this way, the *K*-means++ algorithm and elbow method are applied to overcome the shortcomings of the traditional *K*-means algorithm. The *K*-means++ algorithm is based on the traditional *K*-means algorithm, which makes improvements to the initial clustering center selection; assuming that *n* manufacturing cell centers have been selected (0 < *n* < *K*), then when selecting the first *n* + 1 manufacturing cell centers, the more distant points from the current *n* manufacturing cell center have a higher probability to be selected as the first *n* + 1 manufacturing cell centers. Additionally, it overcomes the effect of random selection of the initial clustering centers of the traditional *K*-means algorithm and effectively improves the clarity and efficiency of manufacturing cell classification [27]. The specific processes of the *K*-means++ algorithm for classifying the manufacturing cells of complex aerospace components are summarized as follows:

  Input: product-equipment matrix *X* = {*x*_1_, *x*_2_, ⋯, *x*_*n*_} and number of manufacturing cells *K*
  Output: *K* manufacturing cells both product processing families *C*_j_ and j = 1,2, ⋯, *n*
  Step 1: we randomly select a sample of points from the product-equipment matrix as the cluster center of the current manufacturing cell *m*_*r*_.
  Step 2: we calculate the shortest distance between each sample and the center of the current manufacturing cell *D*(*x*) = argmin‖*x*_*i*_ − *m*_*r*_‖_2_^2^, where ‖*x*_*i*_ − *m*_*r*_‖_2_^2^ represents the L2-norm of vectors *D*(*x*), that is, the Euclidean distance. On this basis, the probability of each sample point being selected as the center of the next manufacturing cell is calculated: *D*(*x*)^2^/∑_*i*=1_^*n*^*D*(*x*_*i*_)^2^. We select the next manufacturing cell center according to the roulette method.
  Step 3: we repeat step 2 until *K* manufacturing cell centers are selected.
  Step 4: for each sample in the dataset *x*_*i*_, we calculate its distance to the center of the *K* manufacturing cells and classify it in the manufacturing cell with the smallest distance.
  Step 5: for each manufacturing cell *C*_j_, we recalculate its manufacturing cell center *m*_*r*_ = (1/|*m*_*r*_|)(∑_*x*_*i*_∈*m*_*r*__*x*_*i*_).
  Step 6: we repeat steps 4 and 5 until the position of the center of all manufacturing cells no longer produces a change.

To determine the number of manufacturing cells *K*, the elbow method is selected to determine the cluster number *K* based on the construction of product design families. This method calculates the sum of the squared errors (SSE) of the dataset when constructing different manufacturing cells for the current workshop product and equipment situation as shown in equation (1), to judge the merit of the clustering effect. When *K* is less than the true clustering number, an increase in *K* will substantially increase the degree of aggregation of each cluster, so the decrease in SSE will be large, while when *K* reaches the true clustering number, the degree of aggregation obtained by increasing *K* again will rapidly become smaller [28], so the value of *K* corresponding to this inflection point will be called the true clustering number:

(1) $$\begin{array}{c} SSE=\overset{n}{\underset{r=1}{\sum }}\underset{x_{i}\in C_{j}}{\sum }{\left|x_{i}-m_{r}\right|}^{2}, \end{array}$$

where *C*_j_, *x*_*i*_, *m*_*r*_, and SSE refer to the group *j*, the sample points in *C*_j_, the nature heart of *C*_j_, and the clustering error of all samples and reflect the strength of the clustering effect, respectively.

## 3. Model Framework

The model framework is explained in the following sections.

### 3.1. Problem Description

In the multivariety and small batch workshop of the aerospace industry, due to the special characteristics of some movable equipment in aerospace enterprises and the characteristics of multivariety switching of aerospace products, the dynamic reconstruction layout of equipment in the manufacturing cell of the production workshop is needed to cope with the real-time changes caused by the multiproduct switching of typical aerospace complex components on the workshop and to solve the problem of whether the equipment position needs to be adjusted to reconstruct the current layout to ensure the efficiency of the system when the products are switched.

**Figure 3:**
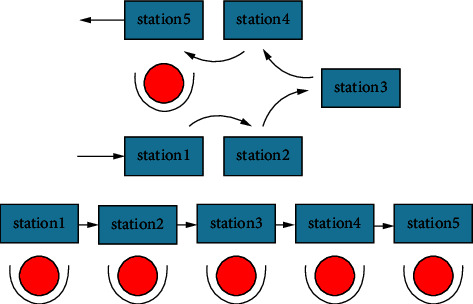
Operation comparison of U-shaped layout and linear layout.

**Figure 4:**
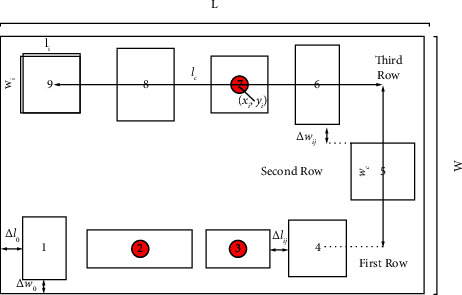
Equipment layout diagram of U-shaped manufacturing cell.

The layout of equipment in the cell mostly uses linear layout or U-shaped layout in actual production as shown in Figure 3. In a cell for multistation continuous operation, compared with the pipeline layout using U-shaped layout, workers move shorter distances, and higher productivity can facilitate the training of multiability workers. Meanwhile, the influence of equipment orientation on the cell area should be considered when designing the cell layout. Therefore, a U-shaped cell reconstruction layout model is established to achieve the minimum of cell logistics cost, reconstruction cost, loss cost, and cell comprehensive area within actual constraints. A schematic diagram of the equipment layout within its workshop manufacturing cell is drawn as shown in Figure 4.

Furthermore, to simplify the problem at hand, we suppose the following. (1) Since equipment used in the workshop is mostly machining centers, we suppose that each piece of equipment is rectangular. (2) Each piece of equipment is arranged in branches in the cell, each row is parallel to the horizontal axis, and the center point of equipment in the same row is located on a horizontal line. (3) Since the center of most equipment coincides with the center of mass, it is assumed that the distance between equipment is the absolute value of the difference between the transverse coordinates of the center of mass and that the logistics are carried out from the center of mass. (4) The material flow between each piece of equipment is constant within the current stage and changes in different stages.

### 3.2. Objective Function

The objective function of the model framework is explained in the following sections.

#### 3.2.1. Material Transport Cost *u*_1_

The calculation of the material transport cost in the cell is the sum of the transport cost per unit distance of the product in each stage, transport times, transport batch, and transport distance:

(2) $$\begin{array}{c} u_{1}=\overset{T}{\underset{t=1}{\sum }}\overset{N}{\underset{i=1}{\sum }}\overset{N}{\underset{j=1}{\sum }}\overset{N}{\underset{k=1}{\sum }}\overset{N}{\underset{l=1}{\sum }}\overset{P}{\underset{p=1}{\sum }}c_{tij}f_{tij}^{p}H_{tp}d_{tij}X_{tik}X_{tjl}\text{,} \end{array}$$

where *X*_*tik*_ and *X*_*tjl*_ decision variables are used to represent the movement of equipment at different stages.

#### 3.2.2. Equipment Reconstruction Cost *u*_2_

The cost of equipment reconstruction mainly involved equipment moving cost and equipment resetting cost which can be calculated by

(3) $$\begin{array}{c} u_{2}=\overset{T}{\underset{t=2}{\sum }}\overset{N}{\underset{i=1}{\sum }}\overset{N}{\underset{k=1}{\sum }}\overset{N}{\underset{l=1}{\sum }}\left(A_{tikl}+S_{tikl}\right)Y_{tikl}\text{.} \end{array}$$

#### 3.2.3. Loss Cost during Equipment Reconstruction *u*_3_

In practice, to guarantee the continuity of production, all equipment are shut down during the equipment reconstruction, so the loss cost is not the profit loss of single reconstruction equipment, but the profit loss of all equipment in the cell during the reconstruction:

(4) $$\begin{array}{c} u_{3}=\overset{T}{\underset{t=2}{\sum }}\overset{N}{\underset{i=1}{\sum }}\overset{N}{\underset{k=1}{\sum }}\overset{N}{\underset{l=1}{\sum }}m_{t}t_{i}Y_{tikl}\text{.} \end{array}$$

#### 3.2.4. Comprehensive Cell Area *S*

To ensure the orderly and safe flow of products between the cells and leave enough space for subsequent track planning, the minimum spacing between cells is required, and equipment in the cell should meet the compactness principle as far as possible. Therefore, the influence of layout dynamics and equipment layout direction on area is considered:

(5) $$\begin{array}{c} S=L\times W=\frac{\overset{T}{\underset{t=1}{\sum }}\left(l_{c}+\max\;\left\{l_{t}^{1}/2,l_{t}^{9}/2\right\}+l_{t}^{5}/2+2\times △l_{0}\right)\times \left(w_{c}+\max\left\{w_{t}^{1}/2,w_{t}^{2}/2,w_{t}^{3}/2,w_{t}^{4}/2\right\}+\max\;\left\{w_{t}^{6}/2,w_{t}^{7}/2,w_{t}^{8}/2,w_{t}^{9}/2\right\}+2\times △w_{0}\right)}{T}\text{.} \end{array}$$

Finally, a multiobjective optimization model of manufacturing cell reconstruction layout for aerospace enterprises is established as follows.

The objective function is

(6) $$\begin{array}{c} \left\{\begin{array}{c} \min\;C=\overset{T}{\underset{t=1}{\sum }}\overset{N}{\underset{i=1}{\sum }}\overset{N}{\underset{j=1}{\sum }}\overset{N}{\underset{k=1}{\sum }}\overset{N}{\underset{l=1}{\sum }}\overset{P}{\underset{p=1}{\sum }}c_{tij}f_{tij}^{p}H_{tp}d_{tij}X_{tik}X_{tjl}+\overset{T}{\underset{t=2}{\sum }}\overset{N}{\underset{i=1}{\sum }}\overset{N}{\underset{k=1}{\sum }}\overset{N}{\underset{l=1}{\sum }}\left(A_{tikl}+S_{tikl}\right)Y_{tikl}\\ +\overset{T}{\underset{t=2}{\sum }}\overset{N}{\underset{i=1}{\sum }}\overset{N}{\underset{k=1}{\sum }}\overset{N}{\underset{l=1}{\sum }}m_{t}t_{i}Y_{tikl}\\ \min\;S=\\ \frac{\overset{T}{\underset{t=1}{\sum }}\left(l_{c}+\max\;\left\{l_{t}^{1}/2,l_{t}^{9}/2\right\}+l_{t}^{5}/2+2\times △l_{0}\right)\times \left(w_{c}+\max\left\{w_{t}^{1}/2,w_{t}^{2}/2,w_{t}^{3}/2,w_{t}^{4}/2\right\}+\max\;\left\{w_{t}^{6}/2,w_{t}^{7}/2,w_{t}^{8}/2,w_{t}^{9}/2\right\}+2\times △w_{0}\right)}{T} \end{array}\right.\text{.} \end{array}$$

Constraints are

(7) $$\begin{array}{c} \overset{T}{\underset{t=2}{\sum }}\overset{N}{\underset{i=1}{\sum }}\overset{N}{\underset{k=1}{\sum }}\overset{N}{\underset{l=1}{\sum }}\left(A_{tikl}+S_{tikl}\right)Y_{tikl}+\overset{T}{\underset{t=2}{\sum }}\overset{N}{\underset{i=1}{\sum }}\overset{N}{\underset{k=1}{\sum }}\overset{N}{\underset{l=1}{\sum }}m_{t}t_{i}Y_{tikl}\leq M\text{,} \end{array}$$

(8) $$\begin{array}{c} \overset{N}{\underset{i=1}{\sum }}X_{tik}=1\text{,} \end{array}$$

(9) $$\begin{array}{c} \overset{N}{\underset{k=1}{\sum }}X_{tik}=1\text{,} \end{array}$$

(10) $$\begin{array}{c} \left|x_{i}-x_{j}\right|\geq \frac{l_{i}+l_{j}}{2}+\Delta l_{ij,} \end{array}$$

(11) $$\begin{array}{c} \left|y_{i}-y_{j}\right|\geq \frac{w_{i}+w_{j}}{2}+\Delta w_{ij}\text{.} \end{array}$$

Equation (7) indicates that the sum of equipment reconstruction cost and loss cost during reconstruction must be less than the budgeted cost. Equation (8) ensures that, at each stage, each location can only accommodate one piece of equipment. Equation (9) depicts that each piece of equipment can only be placed in one position at each stage. Equation (10) guarantees that equipment does not overlap or interfere in the horizontal direction. Equation (11) constrains equipment, so it does not overlap or interfere in the vertical direction.

Note that the dynamic nature of the model is reflected in the change of stage *t*. As the production workshop of the aerospace enterprise is batch production, the logistics amount between each piece of equipment is constant in the current stage, and the production of the next product is started only after the current product batch is all produced, so the production process of the first product of the order is regarded as the first stage *t*_1_ in a manufacturing cell, and when the first product is all produced, it is judged whether it is necessary to adjust the current. If no reconstruction is required, the current layout is retained to enter stage *t*_2_. If reconstruction is required, the product production in stage *t*_2_ starts with the reconstructed layout.

## 4. Proposed Optimization Algorithms

The proposed optimization algorithms are explained in the following sections.

### 4.1. Algorithm Design

U-shape cell reconstruction layout problem has nonlinear and NP-hard characteristics. The complexity of the solution is mainly manifested as the complexity of reconstruction dynamic layout and multiobjective solution which the computational complexity is mainly reflected in the scale of the layout problem. Swarm intelligence optimization algorithms show high performance for solving this problem [7, 12, 13, 29–31]. However, for solving multiobjective dynamic facility layout problem, due to the limitation of equipment location coding, the traditional algorithms such as genetic algorithm and ant colony algorithm may converge slowly, which cannot adapt to the real-time dynamic change of reconstruction layout.

**Figure 5:**
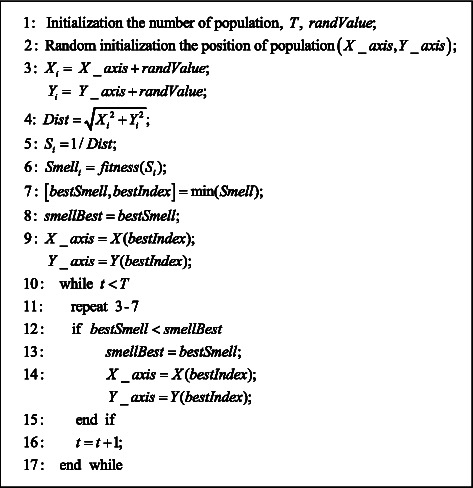
Pseudocode of the FOA.

**Figure 6:**
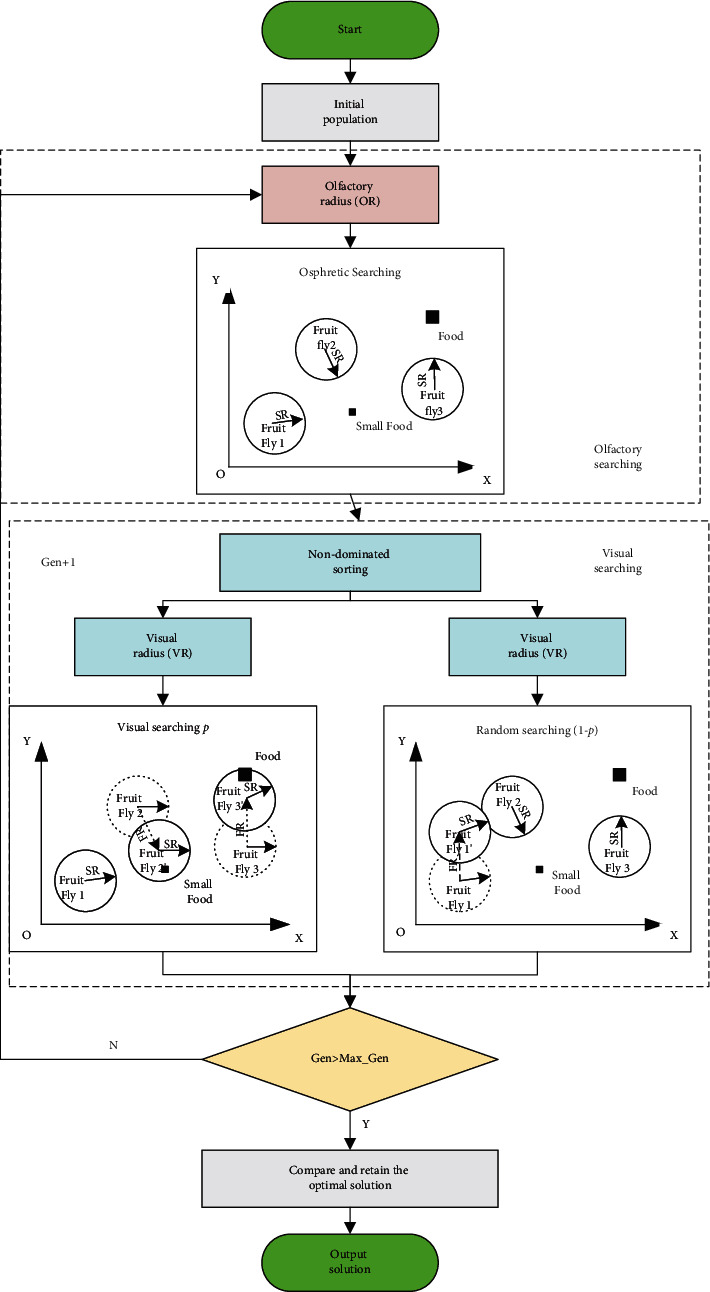
The framework of IFOA.

The fruit fly optimization algorithm proposed by Pan is wildly applied to solve combinatorial optimization problems in recent years, as a result of its strong coding adaptability and fast convergence speed [32]. For solving multiobjective optimization problems, existing studies only combine linear weighting to transform multiobjective problems into single-objective problems, which makes the weight coefficients highly subjective. Therefore, based on the FOA, we introduce the cross-mutation strategy, fast nondominated sorting mechanism, and a random search mechanism based on visual search to improve the stability of the solutions. In this way, the problem of multiobjective dynamic workshop layout is solved; the U-shaped cell of the specific layout of equipment is obtained. The FOA pseudocode is shown in Figure 5, and the flowchart of IFOA is shown in Figure 6.

### 4.2. Encoding Method

**Figure 7:**
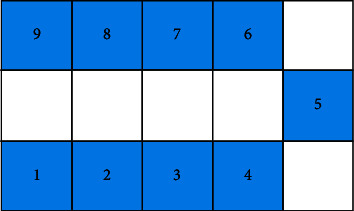
Schematic diagram of encoding of reconstruction equipment layout.

**Figure 8:**
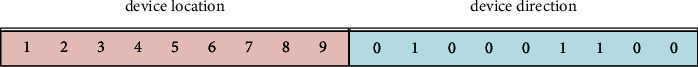
Chromosome coding method.

To satisfy the workshop restrictions and actual needs of the enterprise, equipment in the manufacturing cell in this study follows the U-shaped counterclockwise arrangement principle, which is convenient for workers to operate and can effectively avoid the intersection of logistics. For this reason, for U-shaped manufacturing cell, equipment encoding adopts a mixed encoding method of integer and binary encodings, where the equipment position adopts an integer method, the order is from left to right at the bottom layer, the median is the middle layer, and the top layer is counterclockwise from right to left. In the coding sequence, the center of equipment coincides with the center of the location. For example, one of the nine pieces of equipment in the reconstruction equipment layout is coded as [1–9] as shown in Figure 7. If the number of pieces of equipment is even, a piece of virtual equipment is introduced. The equipment layout direction part adopts (0, 1) binary coding mode, and 0 means that the length of equipment is parallel to the horizontal direction of the cell, while 1 means that the length of equipment is perpendicular to the horizontal direction of the cell. For nine pieces of equipment, an encoding example of their chromosomes is shown in Figure 8. The chromosome adopts this encoding method to directly obtain the specific position of each equipment without decoding.

### 4.3. Define the Olfactory Radius

Randomly, we generate *P* initial populations. The definition of olfactory radius is the exact search step length. In this algorithm, it is expressed as the number of equipment exchanges. The definition OR=2 means that two pairs of equipment bits are randomly selected for exchange. Since the code needs to be a nonrepetitive number, the counterpoint crossing method is adopted.

### 4.4. Olfactory Search and Fast Nondominated Sorting

**Figure 9:**
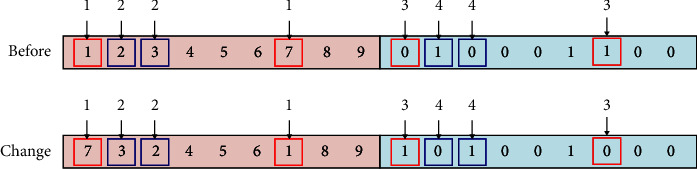
Olfactory search process of IFOA.

Fruit fly gradually approaches food through smell, and substituting new individuals into equation (12) food concentration determination function to calculate food concentration (smell), where *s* represents the individual fruit fly, the equipment sequence, and *F*(*s*) represents the objective function value, which is the minimum logistics cost at the current stage. The greedy method is used to search for a better equipment layout. The olfactory search process is shown in Figure 9:

(12) $$\begin{array}{c} Smell=\frac{1}{F\left(s\right)}\text{.} \end{array}$$

Multiobjective sorting of food concentration values is that, between the cell reconstruction cost and cell comprehensive area, the lower the level, the higher the ranking, and the higher the crowding degree. We select the top fruit fly as the current optimal fruit fly position for subsequent visual search and random search.

### 4.5. Visual Search and Random Search

**Figure 10:**
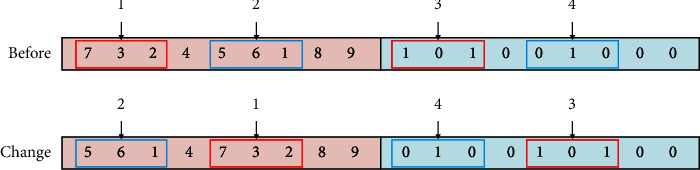
Visual search process of IFOA.

**Figure 11:**
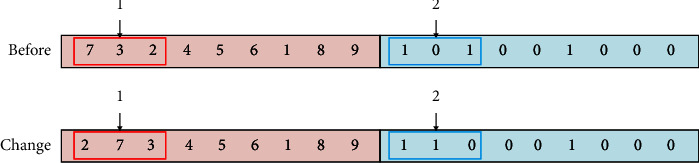
Random search process of IFOA.

Fruit fly approaches food quickly by visual, increases the search step, and sets VR = 3; that is, three consecutive device positions are randomly selected for exchange or variation. The induction probability *p* is introduced in this process. After nondominated sorting, the first *p*% individuals perform a visual search, and the remaining individuals perform a random search. For the visual search, fruit flies approach food quickly through vision and randomly select three consecutive machine tools positions for the entire exchange as shown in Figure 10. In the random search stage, fruit flies fly randomly according to the visual step length to ensure the diversity of the population and prevent it fall into local optimum, that is, randomly select three consecutive machine tools positions for random rearrangement as shown in Figure 11.

## 5. Case Study

**Figure 12:**
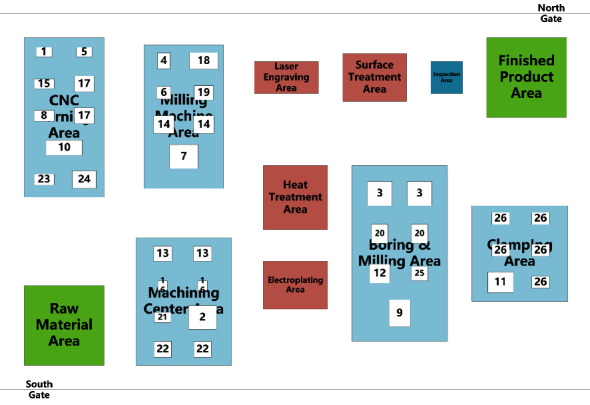
Layout of a typical aerospace production workshop for complex components.

There are many problems in the layout of an enterprise; the more significant ones can be concluded as follows: the average daily logistics volume between computerised numerical control (CNC) boring and milling machining center and electrical discharge machining (EDM) in the clamping area is as high as 25,723 kg, the highest in all manufacturing cell pairs, but the two stations are not close to each other, there is other equipment in between, and the transportation route is as long as eleven meters. The logistics route between the three-axis CNC vertical milling and four-axis boring and milling machining center and the horizontal milling and EDM equipment crosses, and the automated-guided vehicle (AGV) often stops on the route. To cope with the above problems, the layout optimization of complex component production workshop is carried out to verify the feasibility and effectiveness of the proposed method in this study. The aerospace workshop adopts the traditional cluster layout, which consists of twelve areas, namely, raw material area, finished product area, inspection area, heat treatment area, electroplating area, laser engraving area, surface treatment area, CNC turning area, boring machine area, boring and milling area, machining center area, and clamping area, respectively. Enterprise products are mostly military products, statistics in the past three years product orders, and equipment information, the workshop produces twenty-two major products, a total of thirty-seven sets of equipment, and workshop cluster layout as shown in Figure 12.

Through the investigation of the original workshop layout, we found that the workshop has difficulties in workpiece clamping and frequent tooling switching, too much work-in-progress accumulation and low utilization rate of workshop equipment, overlapping logistics routes, and more reverse logistics, which are mainly caused by the continuous increase of product types in the workshop. The original cluster layout of the workshop is no longer suitable for production. Therefore, the enterprise urgently needs to relayout in order to adapt to the dynamic and variable requirements of multi-variety small batch production. At present, the enterprise has purchased some movable equipment to meet the premise of a dynamic layout.

Based on the above analysis, the layout of the aerospace complex component workshop can be optimized from the following directions. Firstly, according to the group principle, the establishment of product parts' family and the centralized production of products with similar process structure can effectively reduce the number of fixtures switching. Secondly, constructing a manufacturing cell can make the logistics cross-concentration within the cell and reduce the occurrence of reverse logistics, thus reducing the overall logistics chaos in the workshop and, finally, concentrating the product in the unit. On the one hand, the flow distance of products is shortened. On the other hand, the utilization rate of unit production equipment and personnel is improved, thus effectively reducing the waiting time of products and further reducing the nonvalue-added time of products.

### 5.1. Product Family Design

**Figure 13:**
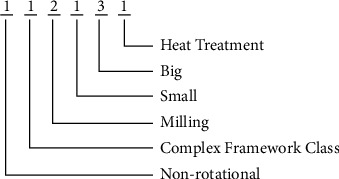
Product family coding rules for aerospace components.

Considering the relatively small number of product types in the production workshops of aerospace enterprises and the special difficulty of representing the structure of typical complex components in aerospace, the feature bit code domain method is applied to encode twenty-two major categories of parts in the workshop, considering the part structure, type, main process, volume, weight, and surface treatment, and constructs the product design family [33]. An encoding example is shown in Figure 13, and the code bit domain values are shown in Table 1.

**Figure 14:**
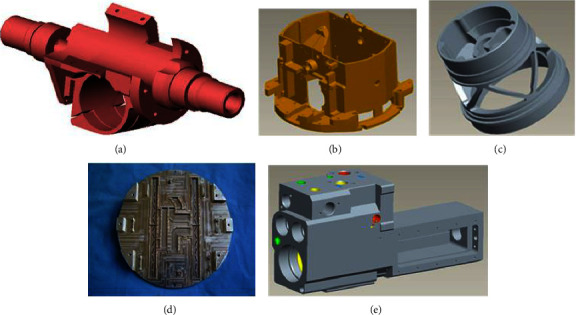
Several types of typical aerospace complex components. (a) Fixed frame structure three-dimensional diagram. (b) Thin-wall shell structure three-dimensional diagram. (c) Guidance structure three-dimensional diagram. (d) Thin-walled plate structure product physical diagram. (e) Cylinder body structure three-dimensional diagram.

As for the previous example, the first bit indicates whether the part is a rotary body. Since 70% of the aerospace components of this enterprise are nonrotational, 0 indicates a rotary body and 1 indicates a nonrotational. The second bit indicates that the part belongs to the type; among them, numbers 1 to 6 represent the complex structure frame class, thin-walled shell class, thin-walled complex structure class, disk shaft class, thin-walled plate class, and channel class, respectively. The third bit indicates the main machining process, and the numbers 1 to 3 indicate turning, milling, and clamping, respectively. The fourth bit indicates the part size class, where 1 indicates *V* ≤ 0.5 m^3^, 2 illustrates 0.5 m^3^ ≤ *V* ≤ 1 m^3^, and 3 refers to *V* ≥ 1 m^3^. The fifth bit indicates part weight class where numbers 1 to 3 demonstrate *W* ≤ 5 kg, 5 kg ≤ *W* ≤ 10 kg, and *W* ≥ 10 kg, respectively. In clustering, the parts with large volume and small weight or small volume and large weight are more likely to be clustered into a class [34]. The sixth position indicates the part surface treatment, and the numbers 0 to 3 represent no treatment required, heat treatment, electroplating, and surface treatment, respectively. Several types of typical aerospace complex components are shown in Figure 14, and the results of constructing product families of typical complex components for twenty-two major categories of aerospace companies are shown in Table 2.

**Figure 15:**
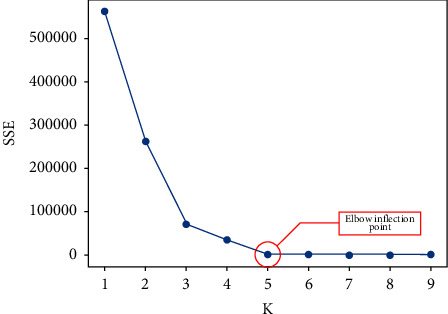
The results calculated by the elbow method.

According to the product design family and process flow used to build the product-equipment matrix shown in Table 3, the matrix has a certain degree of process similarity, but one still cannot intuitively determine which parts should be classified as a manufacturing cell. Therefore, the *K*-Means++ algorithm is performed to determine the precise clustering of parts to divide the manufacturing cell. Firstly, the elbow method is applied to the product design family to determine the clustering *K* value, and the results are shown in Figure 15. From Figure 15, we can see that its elbow inflection point is five; that is, the data in this study are clustered into five classes as the optimal, so *K* = 5 is substituted into the *K*-Means++ algorithm, and its clustering results are shown in Table 4 after finishing.

### 5.2. Dynamic Layout Solution

**Figure 16:**
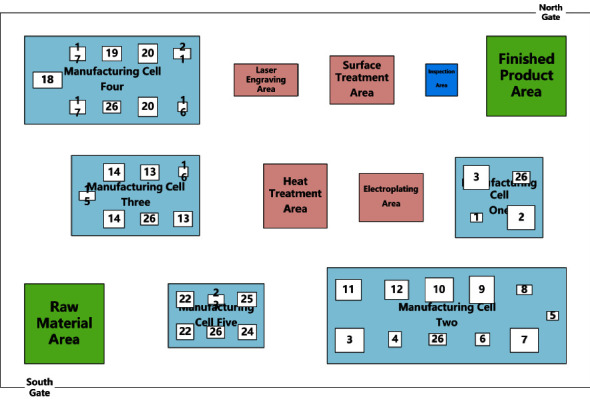
Optimized front cell layout for aerospace complex component production workshop.

The cell layout of the production workshop consists of twelve areas, which are the raw material area, finished product area, inspection area, heat treatment area, electroplating area, laser engraving area, surface treatment area, and five manufacturing cell areas, respectively. Enterprise products are mostly military products, and according to statistics in the past three years for product orders and equipment information, the production workshop produced a total of twenty-two products and a total of thirty-seven sets of equipment, of which the statistical information of each piece of equipment is shown in Table 5. An order cycle of processed products' process route, transport times, and transport batch is shown in Table 6. Among them, the number of transports is determined by the pallet capacity and product orders, independent of the production process route. Based on a comprehensive analysis of the amount of equipment in each manufacturing cell, product types and batches, and product process routes and considering the area of the manufacturing cell, safety distance between equipment, and workers' operation space, the optimized cell layout of the production plant of an aerospace enterprise is drawn based on SLP, as shown in Figure 16. We note that the related information of processing time is available in our previous work [35].

**Figure 17:**
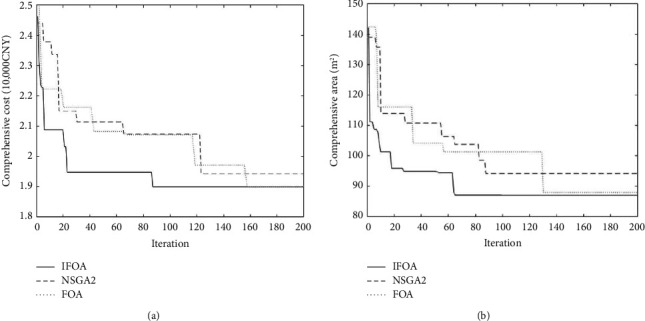
The iteration diagram of (a) comprehensive cost operation and (b) comprehensive area operation.

Taking the manufacturing cell two with the most movable equipment as an example, there are eleven pieces of equipment in its cell, and the processed products are thin-walled shells, cylinder bodies, channel bodies, outer shells, and cases in five categories, and now, the dynamic reconstruction optimization solution is performed for the equipment layout in cell two. Fifty initial layout solutions are randomly generated and brought into MATLAB for iterative operations, including setting olfactory step length OR = 2 and visual step length VR = 3. Decisions are made at each product switch to determine whether to reconstruct the current layout to ensure the efficiency of the system, and the iteration runs for two hundred generations [36]. Moreover, extended experiments between the fast nondominated sorting genetic algorithm (NSGA-II), FOA, and IFOA are conducted. The iteration diagram of comprehensive cost operation and comprehensive area operation are shown in Figure 17.

### 5.3. Experimental Results

After twenty independent runs, the satisfactory solutions are no longer significantly different, and since the cost and area objectives do not conflict with each other, the integrated area can also be optimal if the integrated cost is optimal. Therefore, the segmented solution is carried out in this study, and its calculation results are shown in Table 7, where the results obtained by the original FOA and IFOA indicate that cell two needs to be reconstructed, but the reconstructed solution is not found by NSGA-II and CPLEX. At the same time, the original FOA is more accurate than the NSGA-II, but it tends to fall into the local optimum, resulting in poor convergence. The NSGA-II converges at 122 and 89 generations, respectively, while the original FOA converges only at 158 and 130 generations. The IFOA can jump out of the local optimum more quickly and converge quickly with high accuracy compared with the original FOA as a result of the random search mechanism which enriches population diversity. By comparing the results of IFOA and CPLEX, the comprehensive cost is reduced by 0.66% and the comprehensive area is reduced by 1.13%. It also verifies the correctness of the mathematical model by comparing results obtained by IFOA and CPLEX. The experimental results show that the IFOA outperforms the other three methods in solving multiobjective dynamic facility layout problem.

**Figure 18:**
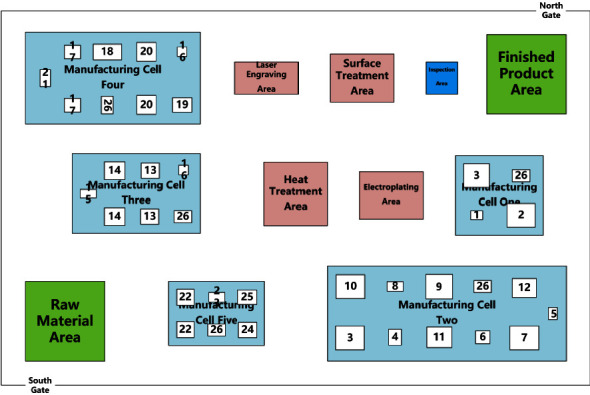
Optimized cell layout for aerospace complex component production workshop.

Furthermore, the IFOA is applied to optimize the layout of each manufacturing cell, and the optimized cell layout is shown in Figure 18. It can be found that manufacturing cell two and manufacturing cell four need to reconstruct the layout during production. On the one hand, manufacturing cell two needs to be reconstructed in the production of the outer shell. And the movable clamp table can be exchanged with the movable milling machine position to reduce logistics costs based on the reason that the outer shell production process uses the clamp table more frequently. On the other hand, manufacturing cell four needs to be reconstructed during wing production. Due to the use of vertical machining center JET40 for wing production, equipment is far from the export of the manufacturing cell; it is necessary to exchange the position with the vertical conversion machining center to reduce the logistics cost.

As shown in Figure 18, after the optimized cell layout, the logistics distance between workstation 11 and workstation 12 is shortened and no longer blocked by other workstations. The total cost of the optimized cell layout is CNY 221,516, of which the logistics cost is CNY 205,774, the reconstruction cost is CNY 11,786, and the loss cost during reconstruction is CNY 3,956. Compared with the logistics cost of the existing cluster layout of the workshop of CNY 242,830, the overall optimization is 8.7%. For this enterprise production workshop cell layout scheme compared with the cluster layout scheme, it can effectively reduce logistics costs; with the aerospace enterprise production workshop, product types continue to increase, the workshop existing cluster layout has gradually become unable to meet the production of multivariety, small batch products, so the workshop cell layout has a great application value.

## 6. Simulation Verification

The production workshop of multivariety and small batch aerospace enterprises is a dynamic discrete system. For its production mode of multivariety and small batch products and the characteristics of a complex production process, the simulation becomes an effective tool to solve the production decision problem of this type of enterprise. Compared with the traditional mathematical analysis, the simulation is more suitable for describing the complex logistics system, and the process and results of the simulation can be previewed at the same time. In the enterprise transformation or new workshop layout before, the application of simulation methods for each planning scheme for virtual operation can make the enterprise personnel understand the effect of the scheme in advance, to achieve the comparison and evaluation between the schemes and timely optimization of the scheme adjustment.

To verify the applicability of the proposed reconstruction layout model and the IFOA, we construct a simulation model of an aerospace enterprise production workshop based on plant simulation, conduct simulation analysis of the before and after optimization scheme, identify simulation entities such as products, equipment, and orders, establish simulation logic by applying SimTalk simulation language, solve the problem of dynamic evaluation of workshop reconstruction layout, and realize the simulation of an aerospace enterprise production workshop.

### 6.1. Simulation Model Establishment

An aerospace enterprise multivariety small batch typical complex components production workshop is taken as the background; the workshop has produced in the past three years a total of twenty-two products and a total of thirty-seven sets of equipment. The workshop is still using the traditional cluster layout, which has a total of twelve areas, respectively: raw materials area, finished products area, inspection area, heat treatment area, electroplating area, laser engraving area, surface treatment area, CNC turning area, boring machine area, boring and milling area, machining center area, and clamping area. After improving the layout of the cells, the workshop has five manufacturing cells, five auxiliary cells, and two storage cells, with thirty-eight workers and five forklifts. At present, single-piece manual transport is used within the cell, and single-piece manual and batch forklift transport are used between cells, where the speed of forklift is 1.2 m/s due to the speed limit.

**Figure 19:**
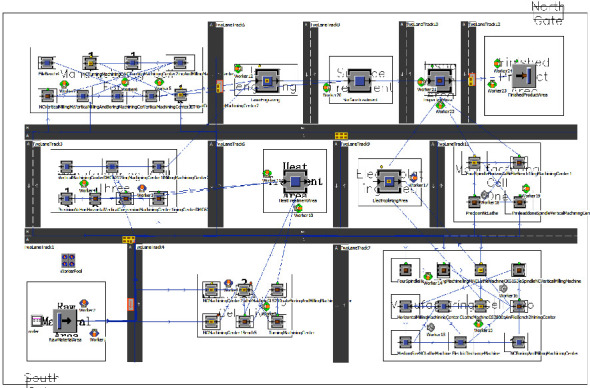
Workshop cluster layout simulation.

**Figure 20:**
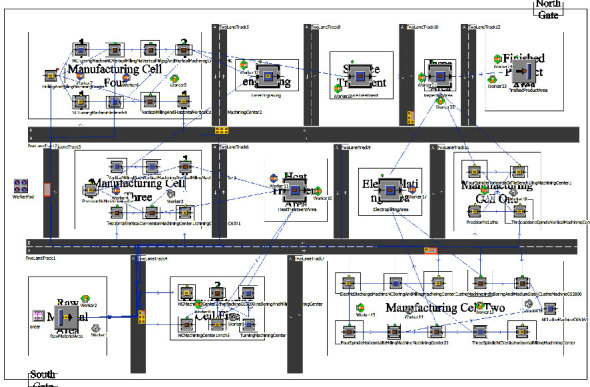
Workshop cell layout simulation.

The aerospace typical complex components production workshop simulation entity is determined according to the above information, where the raw material area uses the source (Source) module for the storage of blank parts. The area of the finished product uses the material end (Drain) module for the storage of finished products. The rest of the equipment is using the processing station (Station) module for the processing of products and inspection; the establishment of the workshop cluster layout and cell layout simulation is shown in Figures 19 and 20.

Orders are the premise of the production workshop simulation, and by setting reasonable product orders, we can compare the advantages and disadvantages of different layout schemes. Since the maximum production capacity of the workshop in a single day is about one hundred sets of components on average, it cannot cover all the products ordered, but the simulation should examine the robustness of the layout; therefore, based on comprehensive product orders in the past three years, all twenty-two kinds of products are brought into the simulation proportionally for a day of production. Simulation of workshop cluster layout and workshop cell layout are carried out separately, and the ratio of their product production, transportation, and storage accounted for and order completion time are counted.

### 6.2. Simulation Output Analysis

The same group of aerospace product orders is brought into the simulation model of cluster layout and cell layout, respectively, and the simulation time is recorded when the source module (Source) generates the first entity, and the forklift transports each blank part to each manufacturing cell, in which the forklift gives priority to the shortest path between two points for distribution, and the shop uses two lanes in both directions to effectively avoid forklift “lock-up.” In the simulation process, all parameters of the cell layout scheme and the cluster layout scheme are set to the same, and only the location of each piece of equipment is considered different.

The simulation ends when the last product of the order is deposited in the material end (Drain), and the summary report of the cluster layout simulation is displayed in the statistical report module. The indexes such as simulation optimization time consumed, average time of a single simulation, and number of simulation evaluations for the case are shown in Table 8 using the above algorithm.

After the model simulation is completed, the ratio of product production, transport, storage, order completion time, and workshop area utilization of each solution are compared to arrive at the optimal cell layout solution.

**Figure 21:**
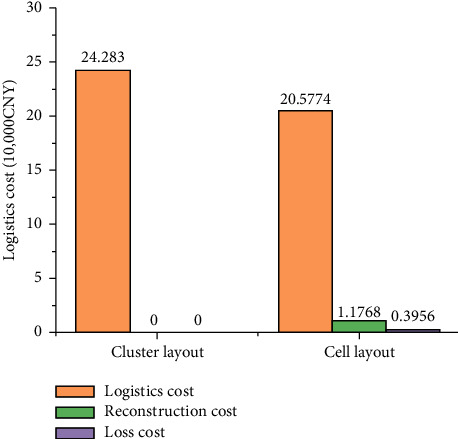
Comprehensive cost comparison.

**Figure 22:**
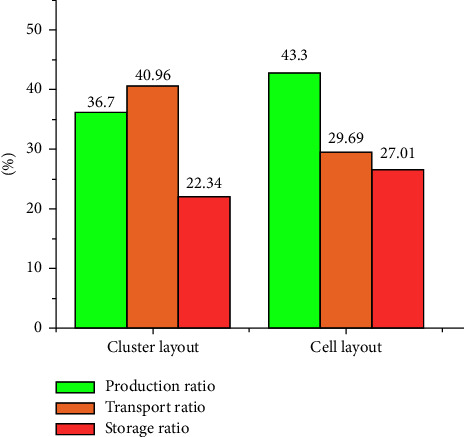
Production-transport-storage comparison.

The calculation method of workshop area utilization is shown in equation (13), the comparison of logistics, reconstruction, and loss costs in the workshop of each scenario is shown in Figure 21, and the comparison of the ratio of production, transport, and storage of workshop products is shown in Figure 22. The comparison of indicators for each scenario is shown in Table 9:

(13) $$\begin{array}{c} \eta =\frac{\overset{12}{\underset{i=1}{\sum }}S_{i}+S_{Tract}}{S_{Total}}\%, \end{array}$$

where *S*_*i*_ is defined as the area of each cell, *S*_Tract_ is defined as the area of tract, and *S*_Toatl_ is defined as the area of total workshop.

Compared with the existing cluster layout, the cell layout can effectively increase the ratio of product production in the typical aerospace complex component production workshop. The use of cell layout can effectively reduce the distance of product transport in the workshop, thus reducing the product transport time, and the product transport is mostly concentrated in the cell, which can realize single-piece flow production in each manufacturing cell, thus effectively reducing the confusion of workshop logistics. At the same time, the similar structure of products in each cell can effectively reduce the number of product mold changes and shorten the production preparation time, thus reducing the product storage time and the number of work-in-process, which verifies the superiority of the U-shaped cell reconstruction layout optimization model proposed in this study and the applicability of the proposed IFOA.

## 7. Conclusion

In this paper, we study the optimization problem of reconstruction layout of multivariety small batch production workshop of aerospace typical complex components.

1. A manufacturing cell layout planning method based on the feature bit code domain method and *K*-Means++ is proposed to realize the accurate division of manufacturing cells.
2. A multiobjective optimization model of manufacturing cell reconstruction layout with the optimization objectives of logistics cost, reconstruction cost, loss cost, and integrated area of the cell is established, and a novel IFOA is presented to solve the model.
3. The better performance of the proposed algorithm has been assessed by the comparison experiments with FOA and NSGA-II.
4. Finally, the simulation models before and after optimization of aerospace complex component production workshop based on plant simulation are constructed. The simulation results show that the optimized workshop cell layout workshop area utilization rate and the available product value-added rate are increased by 5.2% and 6.6%, respectively.

As for the future works, the optimization model of reconstruction layout of multivariety and small batch aerospace typical complex component production workshop is only based on the existing products and process routes of the workshop. If the product types of the workshop are larger enough or the urgent order insertion arises occur, the workshop layout needs to be readjusted. Thus, the followup research will be carried out for the flexibility and robustness of the layout of the workshop. We increase the optimization goal of cell flexibility to achieve the purpose of coping with the dynamic changes of workshop production and consider the robustness of the layout to achieve a comprehensive optimal layout.

## Figures and Tables

**Table 1:** Similarity matrix of aerospace component product family.

Code bit/domain values	I	II	III	IV	V	VI
0	1					1
1	1	1	1	1		
2				1	1	1
3					1	
4			1			

**Table 2:** Aerospace typical complex component product family.

Product family	Part code	Included products
Product family I	112131	Fixation frame, slewing bearing, and rotating frame
	012120	
Product family II	022110	Outside shell, thin-walled shell, waveguide cavity, antenna cavity, and box body
	123121	
Product family III	031110	Steering gear, guidance, baseplate, seat, and center wing box
	132120	
	132221	
Product family IV	141220	Turbine disk and disk shaft
Product family V	152120	Cavity plate, thin-walled plate, antenna panel, wing, and rear wing
	152123	
Product family VI	162112	Channel body and cylinder body

**Table 3:** Product-equipment matrix.

*Equipment model/part name*	*Fixation frame*	*Thin-walled shell*	*Waveguide cavity*	*Cavity plate*	*Rotating frame*	*Seat*	*Antenna cavity*	*Turbine disc*	*Cylinder body*	*Steering gear*	*Thin-walled plate*
Precision CNC lathe	1	0	0	0	0	0	0	0	0	0	0
3 + 1 axis vertical machining center	1	0	0	0	1	0	0	0	0	0	0
Four-axis boring and milling machining center	1	1	0	0	1	0	0	0	0	0	0
Common milling machine	0	1	0	0	0	0	0	0	1	0	0
CNC lathe CK6163	0	1	0	0	0	0	0	0	0	0	0
Three-axis CNC end milling machine	0	1	0	0	0	0	0	0	1	0	0
Horizontal milling machining center	0	1	0	0	0	0	0	0	0	0	0
Medium CNC lathe GS2800	0	1	0	0	0	0	0	0	0	0	0
Five-axis boring and milling machining center	0	1	0	0	0	0	0	0	1	0	0
Medium-sized CNC lathe	0	0	0	0	0	0	0	0	0	0	0
Electrical discharge machining	0	0	0	0	0	0	0	0	0	0	0
NC boring and milling machining center	0	0	0	0	0	0	0	0	1	0	0
Vertical machining center DMC63V	0	0	1	0	0	0	1	0	0	0	0
Vertical milling machining center	0	0	1	0	0	0	0	1	0	0	0
Precision CNC horizontal lathe	0	0	0	0	0	0	1	0	0	0	0
Vertical conversion machining center	0	0	1	1	0	0	1	1	0	0	1
CNC turning center	0	0	0	1	0	0	0	0	0	0	0
Drilling and milling machining center	0	0	0	1	0	0	0	0	0	0	1
CNC vertical milling center	0	0	0	0	0	0	0	0	0	0	1
Vertical milling and boring machining center	0	0	0	0	0	0	0	0	0	0	0
Vertical machining center JET40	0	0	0	0	0	0	0	0	0	0	0
NC machining center	0	0	0	0	0	0	0	0	0	1	0
CNC lathe GLS200	0	0	0	0	0	1	0	0	0	1	0
CNC turning center	0	0	0	0	0	1	0	0	0	0	0
Coordinate boring and milling machining center	0	0	0	0	0	1	0	0	0	0	0
Clamping table	1	1	1	0	1	0	1	1	1	1	1
Precision CNC lathe	0	0	0	0	0	0	0	0	0	1	0
3 + 1 axis vertical machining center	0	0	0	0	0	0	0	0	0	0	0
Four-axis boring and milling machining center	0	0	0	0	0	0	0	0	0	1	0
Common milling machine	0	0	0	0	0	0	0	0	0	0	0
CNC lathe CK6163	0	0	0	0	0	0	0	0	0	0	0
Three-axis CNC end milling machine	0	0	0	0	0	0	0	0	0	0	0
Horizontal milling machining center	0	0	0	0	0	0	0	0	0	0	0
Medium CNC lathe GS2800	0	0	0	0	0	0	0	0	0	0	1
Five-axis boring and milling machining center	0	1	0	0	0	0	0	0	0	0	1
Medium-sized CNC lathe	0	1	1	0	0	0	0	0	0	0	0
Electrical discharge machining	0	1	1	0	0	0	0	0	0	0	0
NC boring and milling machining center	0	0	1	0	0	0	0	0	0	0	0
Vertical machining center DMC63V	0	0	0	0	0	1	0	0	0	0	0
Vertical milling machining center	0	0	0	0	0	1	0	0	0	0	0
Precision CNC horizontal lathe	0	0	0	0	0	1	0	0	0	0	0
Vertical conversion machining center	0	0	0	0	0	0	0	0	0	0	0
CNC turning center	1	0	0	0	0	0	0	0	0	0	0
Drilling and milling machining center	1	0	0	1	0	0	0	0	1	0	0

*Equipment model/part name*	*Antenna panel*	*Outside shell*	*Box body*	*Rear wing*	*Guidance*	*Disk shaft*	*Baseplate*	*Center wing box*	*Wing*	*Slewing bearing*	*Channel body*

CNC vertical milling center	0	0	0	0	1	0	0	0	1	0	0
Vertical milling and boring machining center	1	0	0	1	1	0	0	0	0	0	0
Vertical machining center JET40	0	0	0	1	1	0	0	0	1	0	0
NC machining center	0	0	0	0	0	0	1	1	0	0	0
CNC lathe GLS200	0	0	0	0	0	0	1	1	0	0	0
CNC turning center	0	0	0	0	0	0	1	1	0	0	0
Coordinate boring and milling machining center	0	0	0	0	0	0	1	0	0	0	0
Clamping table	1	1	1	0	1	1	1	1	1	1	1

**Table 4:** Clustering result.

*Equipment model/part name*	*Fixation frame*	*Rotating frame*	*Slewing bearing*	*Thin-walled shell*	*Cylinder body*	*Channel body*	*Outside shell*	*Box body*	*Waveguide cavity*	*Antenna cavity*	*Turbine disk*
Precision CNC lathe	1	0	1	0	0	0	0	0	0	0	0
3 + 1 axis vertical machining center	1	1	0	0	0	0	0	0	0	0	0
Four-axis boring and milling machining center	1	1	1	1	0	0	0	0	0	0	0
Common milling machine	0	0	0	1	1	0	0	0	0	0	0
CNC lathe CK6163	0	0	0	1	0	0	0	0	0	0	0
Three-axis CNC end milling machine	0	0	0	1	1	1	0	0	0	0	0
Horizontal milling machining center	0	0	0	1	0	0	0	0	0	0	0
Medium CNC lathe GS2800	0	0	0	1	0	1	0	0	0	0	0
Five-axis boring and milling machining center	0	0	0	1	1	1	1	0	0	0	0
Medium-sized CNC lathe	0	0	0	0	0	0	1	1	0	0	0
Electrical discharge machining	0	0	0	0	0	0	1	1	0	0	0
NC boring and milling machining center	0	0	0	0	1	0	0	1	0	0	0
Vertical machining center DMC63V	0	0	0	0	0	0	0	0	1	1	0
Vertical milling machining center	0	0	0	0	0	0	0	0	1	0	1
Precision CNC horizontal lathe	0	0	0	0	0	0	0	0	0	1	0
Vertical conversion machining center	0	0	0	0	0	0	0	0	1	1	1
CNC turning center	0	0	0	0	0	0	0	0	0	0	0
Drilling and milling machining center	0	0	0	0	0	0	0	0	0	0	0
CNC vertical milling center	0	0	0	0	0	0	0	0	0	0	0
Vertical milling and boring machining center	0	0	0	0	0	0	0	0	0	0	0
Vertical machining center JET40	0	0	0	0	0	0	0	0	0	0	0
NC machining center	0	0	0	0	0	0	0	0	0	0	0
CNC lathe GLS200	0	0	0	0	0	0	0	0	0	0	0
CNC turning center	0	0	0	0	0	0	0	0	0	0	0
Coordinate boring and milling machining center	0	0	0	0	0	0	0	0	0	0	0
Clamping table	1	1	1	1	1	1	1	1	1	1	1
Classification result	1	1	1	2	2	2	2	2	3	3	3
Precision CNC lathe	0	0	0	0	0	0	0	0	0	0	0
3 + 1 axis vertical machining center	0	0	0	0	0	0	0	0	0	0	0
Four-axis boring and milling machining center	0	0	0	0	0	0	0	0	0	0	0
Common milling machine	0	0	0	0	0	0	0	0	0	0	0
CNC lathe CK6163	0	0	0	0	0	0	0	0	0	0	0
Three - axis CNC end milling machine	0	0	0	0	0	0	0	0	0	0	0
Horizontal milling machining center	0	0	0	0	0	0	0	0	0	0	0
Medium CNC lathe GS2800	0	0	0	0	0	0	0	0	0	0	0

*Equipment model/part name*	*Disk shaft*	*Cavity plate*	*Thin-walled plate*	*Antenna panel*	*Wing*	*Rear wing*	*Guidance*	*Steering gear*	*Baseplate*	*Center wing box*	*Seat*
Five-axis boring and milling machining center	0	0	0	0	0	0	0	0	0	0	0
Medium-sized CNC lathe	0	0	0	0	0	0	0	0	0	0	0
Electrical discharge machining	0	0	0	0	0	0	0	0	0	0	0
NC boring and milling machining center	0	0	0	0	0	0	0	0	0	0	0
Vertical machining center DMC63V	1	0	0	0	0	0	0	0	0	0	0
Vertical milling machining center	1	0	0	0	0	0	0	0	0	0	0
Precision CNC horizontal lathe	1	0	0	0	0	0	0	0	0	0	0
Vertical conversion machining center	0	1	1	0	0	0	0	0	0	0	0
CNC turning center	0	1	0	1	0	0	0	0	0	0	0
Drilling and milling machining center	0	1	1	1	1	1	0	0	0	0	0
CNC vertical milling center	0	0	1	0	1	0	1	0	0	0	0
Vertical milling and boring machining center	0	0	0	1	0	1	1	0	0	0	0
Vertical machining center JET40	0	0	0	0	1	1	1	0	0	0	0
NC machining center	0	0	0	0	0	0	0	1	1	1	0
CNC lathe GLS200	0	0	0	0	0	0	0	1	1	1	1
CNC turning center	0	0	0	0	0	0	0	0	1	1	1
Coordinate boring and milling machining center	0	0	0	0	0	0	0	0	1	0	1
Clamping table	1	0	1	1	1	0	1	1	1	1	0
Classification result	3	4	4	4	4	4	4	5	5	5	5

**Table 5:** Equipment dimensions.

No.	Number	Width (m)	Length (m)
1	1	0.75	0.55
2	1	1.75	1.50
3	2	1.80	1.50
4	1	0.80	1.00
5	1	0.75	0.55
6	1	0.90	0.80
7	1	1.80	1.50
8	1	1.00	0.60
9	1	1.55	1.70
10	1	1.50	0.80
11	1	1.60	1.30
12	1	1.50	1.20
13	2	1.15	0.95
14	2	1.30	1.10
15	1	1.00	0.55
16	2	0.60	0.60
17	2	1.00	0.85
18	1	0.60	0.80
19	1	1.20	0.90
20	2	1.20	1.20
21	1	1.05	0.65
22	2	1.10	1.00
23	1	1.00	0.60
24	1	1.15	0.95
25	1	1.20	0.90
26	5	1.10	0.75

**Table 6:** Product process information.

No.	Process route	Transport times	Transport batch
1	1-2-3-26	4	10
2	2-3-26	3	10
3	1-26-3	3	15
4	3-4-5-26-6-7-8-9	2	10
5	4-6-12-9-26	3	20
6	8-9-26	5	10
7	9-26-10-26-11	3	20
8	10-26-11-12	3	20
9	13-26-16-26-15	4	10
10	13-26-14-16	4	10
11	14-26-16	3	15
12	13-14-26-15	1	20
13	16-17-18	3	10
14	16-18-19-26	3	10
15	17-26-18-20	2	20
16	18-26-19-21	3	15
17	18-20-21	2	10
18	19-26-20-21	3	15
19	22-26-23	2	10
20	22-25-26-23-24	4	10
21	22-23-26-25	3	20
22	23-24-25	3	15

**Table 7:** Comparison results of different methods.

Algorithm	Equipment sequence	Comprehensive cost (CNY)	Comprehensive area (m^2^)
IFOA	[1, 2, 11, 4, 5, 6, 10, 3, 8, 7, 9, 0, 1, 0, 0, 0, 1, 0, 0, 1, 0, 0]	18947	87.6
	[1, 3, 11, 4, 5, 6, 10, 2, 8, 7, 9, 0, 1, 0, 0, 0, 1, 0, 1, 1, 0, 0]		
Original FOA	[1, 2, 11, 4, 5, 6, 10, 3, 8, 7, 9, 0, 1, 0, 0, 0, 1, 0, 0, 1, 0, 0]	18947	87.8
	[1, 8, 11, 4, 5, 6, 10, 3, 2, 7, 9, 0, 1, 0, 1, 0, 0, 0, 1, 1, 1, 0]		
CPLEX	[1, 2, 11, 4, 5, 6, 3, 10, 7, 8, 9, 0, 1, 0, 0, 0, 1, 0, 0, 0, 0, 0]	19073	88.6
NSGA-II	[1, 2, 11, 4, 5, 3, 6, 7, 8, 10, 9, 0, 1, 0, 0, 0, 0, 0, 1, 0, 0, 0]	19425	94.6

**Table 8:** Simulation index comparison table for each scheme.

Optimization indicators	Simulation optimization time consuming (hours)	Average time of single simulation (minutes)	Number of simulation evaluations
Cell layout	28.33	16.8	76
Cluster layout	33.45	25.5	88

**Table 9:** Each scheme index comparison.

	Comprehensive cost	Area utilization (%)	Order completion time	Production ratio (%)	Transport ratio (%)	Storage ratio (%)
Cluster layout	2.3141	70.2	8:59:30	36.70	40.96	22.34
Cell layout	2.0838	75.4	7:39:00	43.30	29.69	27.01

## Data Availability

The data used to support the findings of this study are included within the article.

## Nomenclature

*p*: The product
*t*: The stage
*l*_*i*_: The length of the equipment *i*
*L*: The length of the manufacturing cell
Δ*l*_*ij*_: The minimum distance between equipment *i* and equipment *j* in the same row
*l*_*c*_: The maximum horizontal distance between equipment center points
(*x*_*i*_, *y*_*i*_): The center coordinate of the equipment position
Δ*l*_0_: The distance of equipment *i* in the horizontal direction from the cell boundary
*N*: The total number of equipment sets
*f*_*tij*_^*p*^: The number of times that each batch of products in the manufacturing cell is transported between equipment *i* and equipment *j* in stage *t*
*d*_*tij*_: The transport distance between equipment *i* and equipment *j* in the manufacturing cell in stage *t*
*S*_*tikl*_: The setup cost of moving the equipment *i* from position *k* to position *l* in the stage *t*
*X*_*tjl*_: 1, equipment *j* is at position *l* in stage *t*; otherwise,0
*Y*_*tikl*_: 1, stage *t*, equipment *i* moves from position *k* to position *l*; otherwise, 0
*P*: The total number of products
*T*: The total number of stages
*w*_*i*_: The width of the manufacturing cell
*W*: The width of the manufacturing cell
Δ*w*_*ij*_: The minimum distance between equipment *i* and equipment *j* in different rows
*w*_*c*_: The maximum longitudinal distance between equipment center points
*t*_*i*_: The time required to reconstruct the equipment *i*
Δ*w*_0_: The distance of equipment *i* in the longitudinal direction from the cell boundary
*c*_*tij*_: The transportation cost per cell distance between equipment *i* and equipment *j* in the cell in stage *t*
*H*_*tp*_: The transport batch of product *p* in stage *t*
*A*_*tikl*_: The moving cost of moving equipment *i* from position *k* to position *l* in stage *t*
*m*_*t*_: The production profit obtainable per cell time of the manufacturing cell in stage *t*
*X*_*tik*_: 1, equipment *i* is at position *k* in stage *t*; otherwise, 0.
